# Costing analysis of a Queensland community corrections hepatitis C screening program

**DOI:** 10.1186/s12913-025-13664-y

**Published:** 2025-12-19

**Authors:** Kelly Maynard, Farah Houdroge, Rachael Thompson, Anna L. Hawkes, Alisa Pedrana, Jacqueline A. Richmond, Nick Scott

**Affiliations:** 1 Disease Elimination Program, Burnet Institute, 85 Commercial Rd, Melbourne, Victoria 3004 Australia; 2 Hepatitis Queensland Inc., 12 Birubi St., Coorparoo, Queensland 4151 Australia; 3 School of Public Health, The University of Queensland, 266 Herston Rd, Herston, Queensland 4072 Australia; 4 School of Public Health and Preventive Medicine, Monash University, 553 St Kilda Rd, Melbourne, Victoria 3004 Australia

**Keywords:** Community corrections, Cost, Economic analysis, Hepatitis C, Testing

## Abstract

**Background:**

People involved with the criminal justice system typically have lower healthcare engagement and higher risk of hepatitis C virus (HCV) infection than the general population. Interventions run at community corrections centres have the potential to diagnose and treat previously unreached populations. This study aimed to estimate the cost per treatment initiation and cost per cure through HCV programs at community corrections centres.

**Methods:**

Data were obtained over the period of July 2022 to July 2023 from screening clinics run by Hepatitis Queensland in four community corrections centres located in Brisbane, Australia. All non-drugcosts (presented in 2023 A$ without discounting) were included from the perspective of the clinics, including staff salaries, general practitioner (GP) fees, logistics, medical testing, incentives and overhead costs. Costs of follow up instances were estimated from total nurse follow-up time per clinic.

**Results:**

The total non-drug cost accumulated over 33 clinics in the 13-month study period was $159,982. Among the 185 participants, 23 tested RNA positive (12%). At the end of the study period 8 participants had achieved sustained virologic response (SVR) and 6 were still on treatment. This resulted in a cost per cure of $19,998 or $11,427 per patient initiated on treatment. If GP costs were excluded (i.e. paid by Medicare and not the clinic) the cost per cure and cost per treatment initiation were $12,843 and $7,339 respectively.

**Conclusions:**

As overall prevalence decreases and case finding becomes a greater challenge, providing HCV testing and treatment through community correctional centres may be a cost-effective way of reaching people living with HCV not engaged with healthcare.

## Background

Since the introduction of highly effective direct-acting antivirals (DAAs) for hepatitis C virus (HCV) treatment in 2016, Australia has been working towards the World Health Organization HCV elimination targets of 90% of people with HCV diagnosed and 80% treated by 2030 [1]. The number of people living with HCV in Australia has reduced from an estimated 162,590 in 2015 to 74,400 in 2022 [2]; however, as the number of people living with HCV is reduced, case finding becomes a greater challenge and the costs associated with case finding will increase. Populations with higher risk of HCV, such as people who inject drugs and people in prison, often have lower engagement in HCV-related health care due to challenges with access, stigma and marginalization [3], meaning novel models of care are required to avoid leaving people behind. As such, it is critical to understand which programs and models of care have the greatest testing yields and lowest cost per cure.

People involved with the criminal justice system have long been identified as an at-risk group for HCV infection. This can be attributed to numerous factors including: high rates of injecting drug use, low access to harm reduction services and increased injecting risk behaviours in prison, high re-incarceration rates among people who inject drugs, low healthcare engagement and stigma [3–8]. In Australia, HCV testing and treatment programs have been successfully implemented in prisons. This resulted in 3,414 treatment initiations within the prison system in 2023, 42% of all treatment initiations nationally in that year [9]. However, short sentence lengths and high rates of loss to follow up on release can result in people being left behind. A recent study found that only 25% of people with HCV in prison were successfully linked to care following release and some of these were only re-engaged with care upon re-incarceration [10]. There is a clear need to find other strategies to ensure people who are diagnosed with HCV and being lost from care following release from prison are linked to care and treated.

Moreover, many people involved with the criminal justice system are released on probation or parole and spend little to no time incarcerated. In Australia in 2023, nearly twice as many people were under community corrections orders (*n* = 81,760) as were currently serving prison sentences (*n* = 42,274) [11]. This represents a large group of potentially unreached people who are at high risk of HCV. Community correctional centres have been identified as a valuable candidate for HCV programs to reach additional people living with HCV and accelerate progress towards elimination [12]. Very few studies have been published describing HCV programs at community corrections centres. Winter et al. [12] identified four such studies across the USA and Spain. Community corrections centres in Australia do not generally offer health care services. Therefore, providing HCV testing and treatment in these locations requires a new model of care that will need to include full logistical and staffing costs. Understanding the costs associated with HCV care at community corrections is vital for informing cost-effective models and future investments.

In 2020 Hepatitis Queensland (QLD) initiated a HCV program where regular clinics offered HCV testing and follow up treatment at four community corrections facilities in Brisbane [13]. People attending community corrections and peers in their networks could attend the clinic which involved a general practitioner (GP) consult and free HCV testing with follow up treatment for positive cases. The GP was in attendance specifically for the program. There was no cost to the client for the GP consult. The GP was reimbursed for their time through the project as community corrections is not a Medicare claimable site. All results were followed up by a Community Outreach Nurse and positive cases were offered treatment with prescriptions written by the clinic GP. Follow up monitoring by the nurse continued until treatment completion.

In this paper we aim to estimate the cost from a healthcare providers’ perspective per treatment initiation and cure through the Hepatitis QLD HCV screening program at Brisbane community corrections facilities using a decision tree analysis to map patient pathways through care. In addition, we estimate the proportion of costs attributable to case-finding and loss to follow up.

## Methods

### Clinic overview

In August 2020 Hepatitis QLD began running a pilot HCV screening program at four community corrections centres in Brisbane; a more comprehensive overview of the program over the period of August 2020 – December 2021 is described elsewhere [13]. The program involved a new service where clinics were run at community corrections services. Three to five staff attended each clinic: a Senior Project Officer, Community Outreach Nurse and GP attended every clinic and a Cultural Liaison Officer and QML phlebotomist attended some of the clinics. In addition to clinic attendance, the Senior Project Officer conducted clinic planning, stakeholder engagement and follow up administration. At the clinic, free HCV RNA testing using GeneXpert was offered to people attending community corrections and peers in their networks. Testing was conducted using point-of-care tests and/or QML phlebotomy, along with FibroScan for liver assessments.

After attending a clinic and undergoing HCV testing, patients were followed up by the Community Outreach Nurse with their results, prescriptions were organised by the nurse and written by the GP, the nurse conducted treatment monitoring and an SVR appointment upon treatment completion. Data were recorded on number of clients attending each clinic, RNA status and patient awareness of results (either RNA positive and notified of results, RNA negative and notified of results, or did not receive results), prescribing (either prescription organised and picked up, not prescribed, or further testing/referred to specialist), treatment status (treatment monitoring: still on treatment or completed treatment or unable to be contacted) and treatment completion appointment (SVR appointment attended or missed appointment).

This study focuses on the 13-month period of July 2022 to July 2023, during which time 33 clinics were conducted at four community correctional centres: Redlands, Mt Gravatt, Central Brisbane and South Brisbane.

### Cost analysis

Costs were calculated from the perspective of the outreach clinic, and are presented in 2023 A$ without discounting. Costs were provided by the clinic staff and validated in discussions with the clinic staff.

**Fig. 1:**
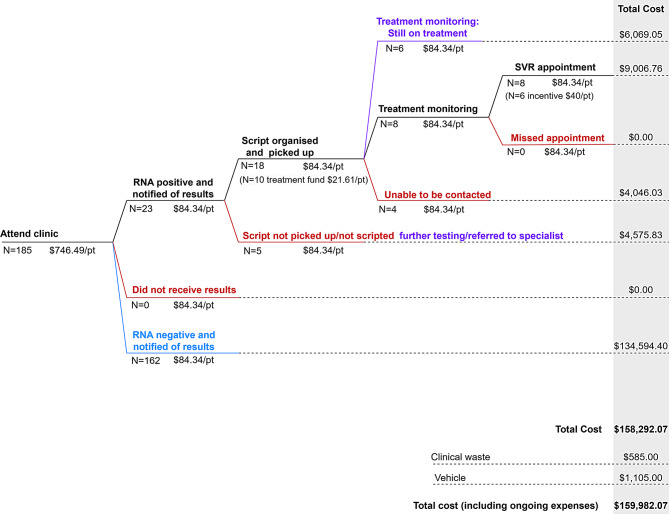
Care cascade stages and costs. Stages of the cascade of care with N patients (pt) at each stage and a cost per patient for each stage. Total costs are shown across all pathways, as well as for all patients on individual pathways

A decision tree was used to map patient pathways through care, with patients able to continue or become lost to follow up (LTFU) at each step between the initial attendance at the clinic and SVR. RNA positive patients were considered LTFU if they were unable to be contacted, were unable to be prescribed or did not pick up their prescription. Each stage in the care pathway was assigned a number of patients based on Hepatitis QLD data (Fig. 1) and a cost per patient (derived below and in Table 1).

**Table 1:** Costs breakdown for clinics, follow up and overhead costs. Costs in bold are included directly in Fig. 1. Costs provided and validated by clinic staff

Item	Number of clinics/clients	Average cost per clinic/client	Total Cost	Source/Notes
**Costs per clinic**				
GP Travel costs	33 clinics	$30	$990	Flat fee
Senior Project Officer	33 clinics	$706.98	$23,330.34	$56.11/hour, 12.6 hours per clinic. Attendance on day plus clinic planning, stakeholder engagement, and follow up admin
Community Outreach Nurse	33 clinics	$246.44	$8,132.52	4-hour clinic time with a salary of $61.61/hour
FibroScan	33 clinics	$500	$16,500.00	Estimated service and maintenance fee per clinic
Meals for project staff	33 clinics	$40	$1,320.00	Lunch provided by HQ for HQ staff, QML staff, GP
Cultural Liaison Officer	21 clinics	$349.92	$7348.32	Aboriginal and Torres Strait Islander Liaison Officer. 6 hours per clinic, $58.32/hour salary. Attended all Central clinics and assumed attended half of all other clinics
GP1 fee	24 clinics	$1,375	$33,000.00	GP attendance at clinic, clinical governance, prescription writing, ongoing patient monitoring with nurse
GP1 Medical surgery fee	24 clinics	$275	$6,600.00	Medical surgery admin staff admin fee
GP2 fee	9 clinics	$1,500	$13,500.00	GP attendance at clinic, clinical governance, prescription writing, ongoing patient monitoring with nurse
GP2 Medical surgery fee	9 clinics	$350	$3,150.00	Medical surgery admin staff admin fee
QML pathology services	18 clinics	$120	$2,160.00	Pathology service screening for HIV, HBV, other liver indicators, reflex HCV testing. Approx. $30/hour, 4 hours/clinic
**Costs per client attending clinic**				
Health care packs	185 clients	$6	$1,110.00	Shampoo, conditioner, toothpaste, toothbrush, soap, condom (all accessed for free by HQ)
Client water/snacks	185 clients	$4	$740.00	Water, mentos, muesli bars etc.
Clinic consumables	185 clients	$5	$925.00	Tissues, large alcohol wipes, rubbish bags.
Incentive vouchers	185 clients	$20	$3,700.00	Clinic attendance
Point-of-care testing consumables	139 clients	$8	$1,112.00	Point-of-care testing cartridges, gloves, blueys, band-aids, minivets, alco wipes, lancets
Clothing	62 clients	$5	$310.00	Provided through Thread Together
Point-of-care testing machine	-	-	$14,172.71	Point-of-care testing machine on loan through Kirby National point-of-care testing program. Total cost for study period estimated based on machine rental cost, delivery and accessories assuming a two-year rental period.
**Total clinic costs** (average per clinic)	33 clinics	$4,184.88	$138,101.09	
Average per client	185 clients	**$746.49**		
**Post clinic costs**				Assuming 8.6 hours follow up time per clinic with a salary of $61.61/hour. Follow up time estimated in consultation with clinicians. Total of 234 follow up events assumed to have equal time spent on each event
Nurse follow up costs	234 events		$17,484.92	
GP2 Surgery room hire	9 clinics	$250	$2,250.00	Use of GP surgery room for Hepatitis C clinic patient follow up
**Total follow up costs**	234 events	**$84.34**	$19,734.92	
Treatment fund	10 clients	**$21.61**	$216.10	
SVR incentive	6 clients	**$40.00**	$240.00	Since January 2023 additional $40 voucher incentive to complete SVR4.
**Overhead costs**				
Clinical waste bin			**$585.00**	13-month period, $45/month
Vehicle cost			**$1,105.00**	13-month period, 0.85 cents/km, 100 km/month
**Overall program cost**			**$159,982.07**	

The average cost per patient for the initial clinic visit was calculated from the total clinic costs (across the 33 clinics) divided by the 185 patients who attended. Clinic costs included: staff time (Community Outreach Nurse, GP, Senior Project Officer, Cultural Liaison Officer), point-of-care testing machine and/or QML pathology, FibroScan, clinic consumables, incentives and other health care items provided to clients.

Several follow up events (being notified of results [*N* = 23 positive + 162 negative], having treatment prescription organised [*N* = 18 picked up + 5 not picked up], being monitored on treatment [*N* = 8 completed + 6 still on treatment + 4 not able to be contacted], SVR appointment [*N* = 8]) included nurse follow up time and room hire at one location for follow up purposes. Each follow up event was assumed to have the same cost per patient, calculated as total follow up costs divided by the number of follow up instances (*N* = 234). Total follow up costs consisted of nurse time allocated for follow up and room hire at some locations.

Other costs included in the analysis were the cost of patients accessing the treatment fund (to cover prescription dispensing costs) (*N* = 10) and SVR incentives (financial reimbursement for returning for SVR testing) (*N* = 3), and overhead costs (added to the final total). GP prescribing costs were included in the flat fee for GP services at the clinic (paid for through the project) and were not able to be separated into a follow up cost.

Drug costs were excluded because this analysis took the perspective of the outreach clinic and they are covered by the Pharmaceutical Benefits Scheme (PBS), and also because treatment is the main outcome of this study (hence we only consider care cascade costs to achieve the outcome of treatment).

The cost per cure was estimated as the total cost for all expenses divided by the number of people achieving SVR. Cost per patient initiated on treatment included patients completing SVR and patients still undergoing treatment at the end of the study period.

To explore options for making the model more cost-effective, a sensitivity analysis was conducted by considering the cost per cure if GP costs were not included, to explore what a model may look like that utilized an existing GP site for prescribing where services could be charged to Medicare Benefits Schedule (MBS) items, rather than paying for GP time as was done within this model. An additional sensitivity analysis was performed by considering the cost breakdown when follow up instances among RNA positive patients were assumed to have twice the cost of follow up calls with results for RNA negative patients for each individual follow up event.

## Results

The total cost of the community corrections HCV program was $159,982 for the 13-month period (Fig. 1). Of this total, 9% ($15,076) of costs were spent on patients who initiated treatment, 5% ($8,622) on RNA positive patients who were LTFU, 84% ($134,594) on people who tested RNA negative and 1% ($1,690) on overhead costs.

During the 13-month period, 33 clinics were run with a total of 185 participants. Among the 23 participants who tested RNA positive (12% positivity), 8 patients completed treatment and achieved SVR, 6 patients were still on treatment at the end of the study period and 9 patients were LTFU or unable to be prescribed.

This resulted in a cost of $19,998 per cure (for patients completing SVR), or $11,427 per patient initiated on treatment.

## Sensitivity analysis

If GP costs (36% of total costs) were excluded, the cost per cure was $12,843 and the cost per patient initiating treatment was $7,339. When follow up instances among RNA positive patients were assumed to be twice as expensive as follow up instances among RNA negative patients the proportion of total costs spent on people who tested RNA negative decreased from 84% to 82%.

## Discussion

To our knowledge this study represents the first economic evaluation of an HCV community corrections program in Australia. The non-drug cost per person initiating treatment was $11,427 ($7,339 per patient if GP costs are excluded).

The majority of costs accrued by the program were for case finding, with 84% of costs attributed to people who were RNA negative. Only 9% of costs were spent directly on RNA positive patients initiating treatment; which reflects the past success of Australia’s HCV elimination response and the resulting declining HCV incidence and prevalence. Costs attributed to RNA positive people who were LTFU accounted for 5% of total expenses. This model only includes costs associated directly with the program and does not consider costs associated with re-engaging people who have been LTFU and linking them to treatment. Therefore, it underestimates the true costs of people LTFU. Conversely this study also does not capture individuals LTFU who were later re-linked to care in other locations and may underestimate some of the benefits of the program in diagnosing these individuals. The low proportion of expenses spent on people LTFU in this model is also due to the relatively high rates of retention in care (61%) which could be a result of the large investment in nurse follow up time $19,734.92 (12% of total costs). Additional increases to retention in care would lead to further valuable reductions in cost per treatment initiation.

Staffing costs accounted for a high proportion of the costs for this program with GP costs making up 36% of total costs. If GP fees were able to be covered through the MBS within a similar program it could substantially lower the cost per patient initiated on treatment. When GP costs were excluded the cost per patient initiation on treatment reduced from $11,427 to $7,339. Care would need to be taken not to reduce other staffing costs where staff time played a core role in the benefits of the program, particularly their role in increasing community engagement and linkage to care.

A similar study at primary health services in Australia found that the cost per treatment initiation through community-based testing campaigns prioritising people who inject drugs was $5,878 [14]. This is lower than the cost per person initiated on treatment of $11,427 (including GP costs) and $7,339 (excluding GP costs) obtained in this study. The differences in cost are in part due to higher cost estimates for the FibroScan machine for the community corrections programs as it was required for longer. Additionally, costs for nurse follow up time at community corrections were higher due to differences in nurse wages and higher nurse follow up time, which may also have contributed to increased retention in care (61% through this community corrections program vs 42% through the community-based testing campaign) [14]. Providing HCV testing and treatment via a service that does not usually provide healthcare will naturally accrue higher costs than “adding on” HCV testing/treatment to an existing healthcare service. The observed 12.4% test positivity rate suggests that the community corrections program was also reaching people at high risk of HCV, and provides some justification for this model.

High rates of re-incarceration in combination with strong testing and treatment programs in prisons provides opportunities for people involved with the justice system to access HCV care. For example, in Queensland in 2022–2023 40% of adults released from prison returned to prison with a new sentence within two years [15]. Despite this opportunity to provide HCV treatment, re-infection incidence in prison is high (estimated to be 12.5 per 100 person-years in a New South Wales, Australia study [16]), meaning that people may still leave prison with HCV. Moreover, many people attend community corrections without spending time in prison. From 2005 to 2019 in Queensland, there were 142,803 community-based orders, and in Victoria, Australia, 62% of community corrections orders were not combined orders (i.e. did not include a prison sentence) [17, 18]. Therefore, this program has capacity to reach a group of people who will either not be reached by current prison services or may be LTFU after release from prison.

It is challenging to estimate the cost of not identifying and treating people with HCV through community-based models of care. A 2016 study estimated the lifetime cost of an untreated person with HCV to be around $22,000 [19]. However, this estimate is from before DAAs, and in the current paradigm of broad DAA access if someone is not treated through a community-based model of care they are unlikely to continue liver disease progression indefinitely without their HCV being diagnosed and treated elsewhere.

This study has some limitations. The costing was undertaken from the perspective of the outreach clinic, and did not include administrative and management overhead costs (e.g. reporting, management, IT and communications, printing, office consumables). These costs would be important for considering scale-up, as they may increase total program delivery costs by over 20%. The study also assumed equal costs across all types of follow up (being notified of results whether positive/negative, having treatment prescription organized whether picked up or not, being monitored on treatment, SVR appointment); this does not impact the cost per treatment initiation or cost per cure, but the proportion of costs attributable to people who were HCV-positive/negative. Sensitivity analyses showed that different assumptions made little overall difference. There were some benefits not captured, including reductions in incidence among people who inject drugs through treatment-as-prevention [20]. It is unknown whether the clients reached in this study are engaged in care through other programs such as GP clinics or needle and syringe programs. However, as people with a history of incarceration often face additional challenges and competing priorities which prevent them from accessing healthcare services [21, 22], this program could be instrumental in testing and treating people who would otherwise remain unreached. A strength of this study is that is based on data directly from an actual pilot study.

## Conclusions

Across the 13-month study period, the 33 Hepatitis QLD community corrections HCV clinics tested 185 people, of which 23 (12%) were HCV-positive, and at the end of the study period 8 had achieved SVR and 6 were still on treatment. The total cost was $159,982, with a cost per cure of $19,998 or $11,427 per patient initiated on treatment ($12,843 and $7,339 respectively if GP costs were excluded). As HCV prevalence declines and case finding becomes more expensive, HCV screening programs run through community correctional centres may be a cost-effective strategy for finding and treating remaining people living with HCV on the path to elimination.

## Data Availability

All data generated or analysed during this study are included in this published article.

## Abbreviations

HCV: Hepatitis C virus
DAAs: Direct-acting antivirals
QLD: Queensland
GP: General practitioner
SVR: Sustained virologic response
LTFU: Lost to follow up
PT: Patients
